# Does hyaluronic acid affect hard tissue healing in alveolar bone augmentation procedures? A systematic review of controlled clinical trials

**DOI:** 10.1007/s00784-025-06674-x

**Published:** 2026-02-26

**Authors:** Lukas Bayyigit, Danijel Domic, Christian Ulm, Kristina Bertl, Andreas Stavropoulos

**Affiliations:** 1 Department of Oral Surgery, University Clinic of Dentistry, Medical University of Vienna, Sensengasse 2a, 1090 Vienna, Austria; 2 Department of Periodontology, Dental Clinic, Faculty of Medicine, Sigmund Freud University, Freudplatz 3, 1020 Vienna, Austria; 3 Department of Periodontology, Blekinge Hospital, Hälsovägen, Byggnad 13, 371 41 Karlskrona, Sweden; 4 Periodontology, Faculty of Odontology, University of Malmö, Carl Gustafs Väg 34, Malmö, 205 06 Sweden; 5 Department of Periodontology, School of Dental Medicine, University of Bern, Freiburg Str. 7, 3010 Bern, Switzerland; 6 Department of Conservative Dentistry and Periodontology, University Clinic of Dentistry, Medical University of Vienna, Sensengasse 2a, 1090 Vienna, Austria

**Keywords:** Alveolar bone augmentation, Alveolar ridge preservation, Guided bone regeneration, Hyaluronic acid, Maxillary sinus floor augmentation, New bone formation

## Abstract

**Objective:**

To provide an overview on the effect of hyaluronic acid (HyA) on hard tissue healing in alveolar bone augmentation procedures in humans (PROSPERO registration: CRD42023464863).

**Methods:**

Three databases were searched until April 2025. Studies using HyA in any form and in any alveolar bone augmentation procedure were included, if a control group allowing to assess the effect of HyA was available; studies addressing periodontal regeneration or using HyA coated implants were not included. Primary outcome parameters, summarized descriptively, were histologic/radiographic new bone formation (NBF), alveolar ridge width (ARW) and height (ARH). The Cochrane Collaborations RoB 2.0 and ROBINS-I tool were used to assess the risk of bias.

**Results:**

Fourteen studies with a follow-up of 3–12 months were included, which contributed with 229 patients and 317 sites allowing to assess the effect of HyA. HyA was assessed as adjunct to maxillary sinus floor augmentation (MSFA), guided bone regeneration (GBR), and alveolar ridge preservation (ARP). The addition of HyA significantly improved NBF in 50 and 66% of the MSFA and ARP studies, respectively; however, a significant clinical improvement was not consistently observed. Advantages for NBF as well as ARW or ARH gain have been reported in GBR procedures, but the available data are limited and mostly descriptive. No differences were observed regarding reported post-operative complication rates. Due to the heterogeneity in treatments and outcome measures, as well as the limited number of studies, a meta-analysis was not feasible for any of the outcomes.

**Conclusions:**

In the original studies, positive and occasionally significant results have been reported for the adjunct use of HyA in alveolar bone augmentation; however, the effect size and clinical relevance is unclear, and further clinical research is needed.

**Clinical relevance:**

HyA in alveolar bone augmentation is safe, but it remains unclear in which indication and in which application form it may enhance quality and/or quantity of NBF and at what extent.

## Introduction

Alveolar ridge dimensions are significantly reduced after tooth extraction, amounting to an average horizontal bone loss of 2.5 to 4.6 mm and vertical bone loss of 0.9 to 3.6 mm after 3 to 12 months of spontaneous healing [1]. Implant installation is frequently chosen to replace missing teeth, which can be performed at different timepoints after tooth extraction, i.e., either immediate (on the same day), early (after 4 to 8 weeks), delayed (after 3 to 4 months), or late (after > 6 months) [2]. Independent of the timepoint, implants should be placed in a prosthetically driven position, which may require hard tissue augmentation to ensure an adequate bone volume surrounding the implant, i.e., at least 1.5 mm are recommended in the buccal and palatal/lingual direction [3]. Various techniques are available to either reduce alveolar bone loss or to regenerate the lost bone, either prior or simultaneously to implant installation. In particular, alveolar ridge preservation (ARP) attempts to reduce alveolar bone resorption occurring after tooth extraction, e.g., by applying bone substitute materials (BSM) into the extraction socket [4, 5], while an already existing horizontal and/or vertical bone deficiency can be corrected by guided bone regeneration (GBR) [4, 6–10] or specifically in the posterior maxilla by maxillary sinus floor augmentation (MSFA) [4, 11–13].

Commonly, hard tissue augmentation procedures include the use of autologous bone, allografts, xenografts, alloplasts, or combinations thereof [4, 14–17]. Furthermore, to enhance the self-regenerative capacity of the jawbone [18], accelerate the healing process, improve the outcome, and/or reduce complication rates of hard tissue augmentation procedures, the adjunct use of “biologics”, e.g., recombinant human bone morphogenetic protein-2 (rhBMP-2), enamel matrix derivates (EMD), or blood-derived products, has been broadly assessed. The idea behind these biologics is to positively affect various cellular pathways of the wound healing process, enhancing angiogenesis, osteogenesis, and/or extracellular matrix formation. Although the adjunct use of biologics has been described to improve histomorphometric outcomes, such as new bone formation (NBF), the limited currently available evidence does not indicate significantly improved clinical and radiographic outcomes over control groups without biologics; this observation was so far quite irrespective the clinical indication [19].

**Fig. 1:**
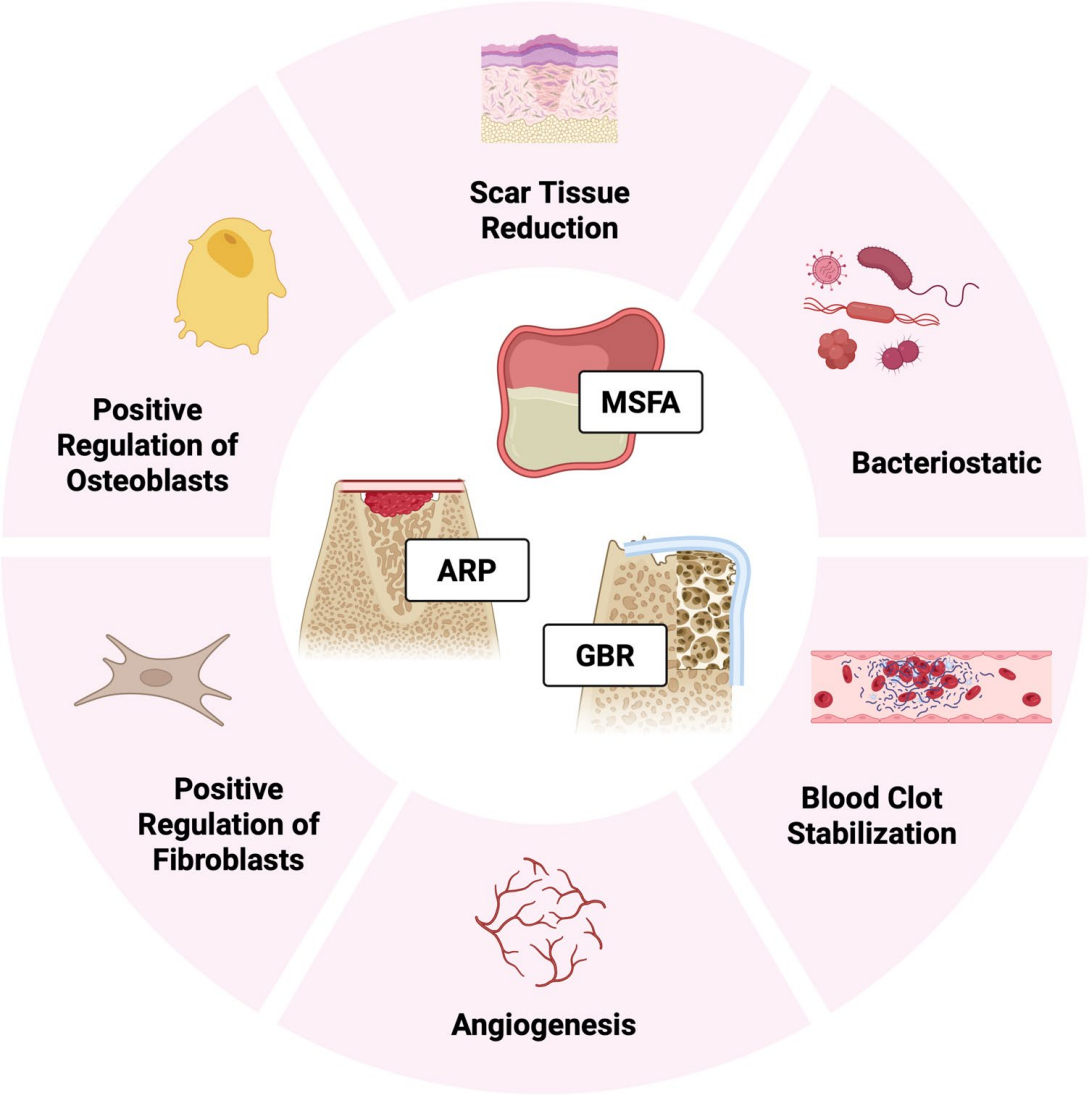
Overview of the mechanisms of action of HyA, which could contribute positively to alveolar bone augmentation procedures; the information for this figure was derived from: [35–40]. Abbreviations: ARP—alveolar ridge preservation; GBR—guided bone regeneration; MSFA—maxillary sinus floor augmentation. Created in BioRender. Domic, D. (2025) https://BioRender.com/i9wx5si

Hyaluronic acid (HyA) – another biologic – is a glycosaminoglycan naturally occurring in the human body. It plays a role in many processes, e.g., it mediates inflammation and enhances soft and hard tissue healing [20, 21]. In in vitro studies the stimulation of mesenchymal stem cells with HyA lead to a significantly higher expression of growth factors, such as rhBMP-2, fibroblast growth factor (FGF), and vascular endothelial growth factor (VEGF) [22]. Further, in preclinical in vivo trials the application of HyA as an adjunct to different bone substitute materials (BSM), which were applied into bone defects in the mandible [23], distal femur [24], or calvaria [25], increased the activity of osteoblasts as well as the expression of osteogenic markers, leading to a superior rate of NBF compared to the groups without adjunct use of HyA. Figure 1 summarizes the mechanisms of action of HyA, which could contribute positively to alveolar bone augmentation procedures. In clinical trials, the use of HyA alone or with a carrier appeared to improve post-operative morbidity after surgical removal of lower wisdom teeth and potentially the soft tissue healing after non-surgical tooth extraction; however, the data – especially on the latter topic – are still inconclusive [26]. Furthermore, a recent systematic review comparing histomorphometric results after bone augmentation in humans using BSM with or without HyA showed no significant benefit from the application of HyA; however, only 3 studies on rather different clinical indications contributed to the meta-analysis included in the study, i.e., MSFA and ARP [27]. Another systematic review on the effect of HyA specifically in ARP procedures concluded that HyA combined with a BSM might improve bone regeneration and graft stabilization, and reduce graft resorption [28]. Nevertheless, the evidence on the effect of HyA on hard tissue healing in clinical studies remains inconclusive [29–34].

Therefore, the present systematic review aimed to provide a broad overview on the effect of HyA application on hard tissue healing in alveolar bone augmentation procedures. More specifically, this systematic review addressed the following PICOS question: „In patients requiring alveolar bone augmentation, does the application of HyA alone or in combination with other products/carriers result in superior alveolar bone regeneration (histologically, radiographically, or clinically) and/or reduced complication rates compared to a control treatment? “

## Material and methods

### Study protocol and registration

The present review followed the Preferred Reporting Items for Systematic Reviews and Meta-Analyses (PRISMA) 2020 guidelines for performing systematic reviews [[41], (Appendix 1)] and the protocol was registered in PROSPERO (CRD42023464863).

### Information sources, literature search, and eligibility criteria

The literature search was performed in 3 databases (Ovid (MEDLINE and CENTRAL), EMBASE, and Pubmed) and last updated on the 18th of April 2025. Details on the search including keywords are presented in Appendix 2. After removing duplicates, the titles and abstracts were screened for eligibility by 2 reviewers (LB, DD) and any ambiguity was resolved by discussion with a third author (KB). Kappa-values were calculated for full-text screening and final inclusion of the studies. After the literature search was completed, a screening of the reference lists of the included studies and available reviews, as well as a forward search, were performed.

The following eligibility criteria were applied in the screening process: 1) written in English language, 2) full-text available, 3) availability of clinical, radiographic, and/or histological data, 4) prospective controlled clinical trials (CT) and randomized controlled clinical trials (RCT) with 5) ≥ 3 months of follow-up and 6) at least 10 treated sites in total, 7) any type of alveolar bone augmentation (e.g., MSFA, ARP, horizontal or vertical GBR, etc.), where 8) HyA was applied directly into the defect, used as a coating for or mixed with autologous bone, BSM, any type of scaffold, and/or covering a membrane, or topically applied, and 9) availability of a control group allowing to assess the pure effect of HyA. Specific exclusion criteria were 1) the assessment of the effect of HyA in periodontal regeneration, 2) HyA used as a coating for implants, and 3) case reports/series.

### Data collection and extraction

The data from the included studies were extracted by one reviewer (LB) and double-checked by 2 other reviewers (DD, KB). The following data were extracted from each study: (1) first author, (2) type of intervention, (3) publication year, (4) study design, (5) patient characteristics (gender, age, health-, and smoking status), (6) inclusion criteria, (7) number of treated sites, (8) treatment groups, (9) follow-up period, (10) postoperative medication, (11) available outcome parameters, and (12) outcome data. Furthermore, all available details regarding the applied HyA products were summarized: (1) trade name, (2) manufacturer, (3) concentration (4) application-, and (5) chemical form.

### Risk of Bias (RoB) assessment

For RCT the Cochrane Collaborations RoB 2.0 Tool was used [42]. The following criteria were evaluated as having „low “, „some “, or „high “ concerns for RoB: (1) randomization process, (2) deviations from intended interventions, (3) missing outcome data, (4) measurement of the outcome, (5) selection of the reported result, and (6) overall RoB. The non-randomized trials were evaluated with the ROBINS-I tool [43]. The RoB was judged as „low “, „moderate “, „serious “, „critical “, or „no information “ for the following criteria: (1) confounding, (2) selection of participants, (3) classification of interventions, (4) deviations from intended interventions, (5) missing outcome data, (6) measurement of the outcome, (7) selection of the reported result, and (8) overall RoB. The assessment was done by one reviewer (LB) and verified by 2 other reviewers (DD, KB).

### Synthesis of results and statistical analysis

Pair-wise meta-analysis was planned in case of at least 3 RCT with similar study design (i.e., treatment indication, HyA regime, follow-up period, and outcome assessment) to assess the pure effect of HyA. Histological NBF, alveolar ridge height (ARH), and alveolar ridge width (ARW) were defined as primary outcome parameters. Secondary outcome parameters were the percentage of connective tissue and residual graft material, and post-operative complications including patient reported outcome measures (PROM).

## Results

### Study selection and characteristics

The literature search is presented as flowchart in Appendix 3. In total, 2644 studies were identified through database search, and 6 additional studies through “manual” search. After removal of duplicates, 1572 studies remained; after initial screening of title and abstract, the full-texts of 27 studies were assessed and 14 studies were finally included [29–34, 44–51]. Both reviewers fully agreed on the studies chosen for full-text screening (kappa = 1) and almost perfectly for the studies finally included (kappa = 0.897). The reasons and references for the 13 excluded studies are listed in Appendix 4.

Twelve of the included studies were RCT [30–34, 44–49, 51] and 2 were CT [29, 50]; 4 RCT were performed in parallel group design [31, 33, 34, 49] and 8 in split mouth design [30, 32, 44–48, 51]. All included studies could be allocated to one of the following 3 surgical procedures: MSFA (n = 7) [29–31, 44–47], ARP (n = 5) [32, 33, 48–50], and GBR (n = 2) [34, 51].

### Study population

In the MSFA, ARP, and GBR studies a total of 101, 107, and 21 patients, respectively, were included, providing 83, 68, and 20 test sites receiving HyA as adjunctive treatment. However, only 67, 68, and 20 of these sites allowed the assessment of the effect of HyA in a direct comparison to 67, 68, and 27 control sites without HyA; 16 MSFA sites received also HyA as adjunctive treatment but in combination with different BSM compared to the available control group. In all studies, patients were either healthy or had no significant or advanced systemic disease. While 3 studies did not report the smoking status of the patients [30, 34, 50], 7 studies included only non-smokers [29, 32, 33, 46–49], one study each included patients smoking ≤ 10 [31] or ≤ 15 cigarettes/day [51], respectively, and 2 studies included both smokers and non-smokers [44, 45]. All details of the included studies are presented in Table 1.

**Table 1.**
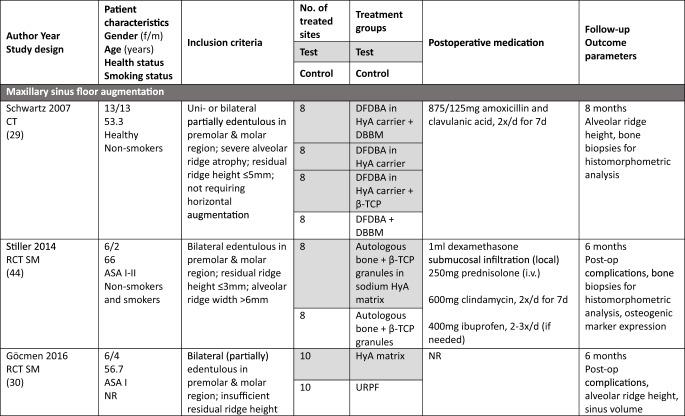

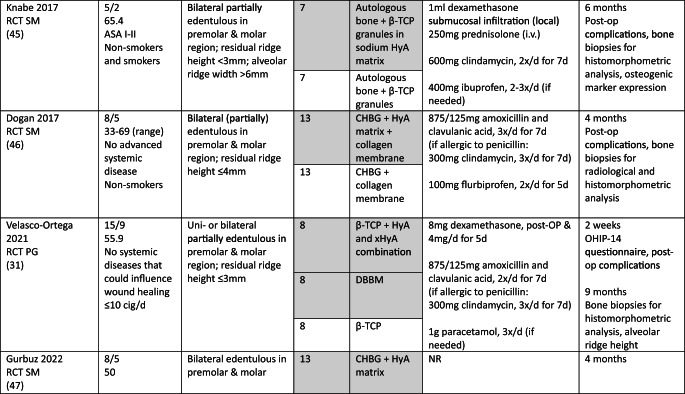

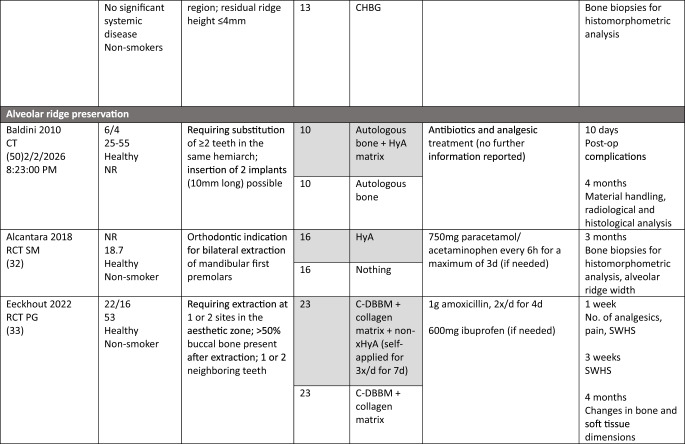

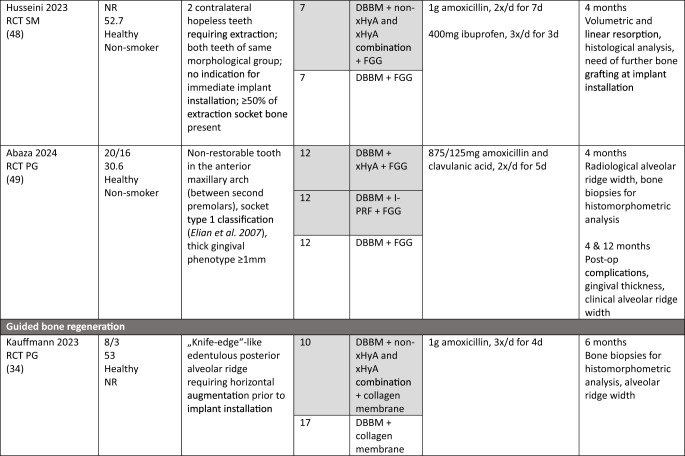

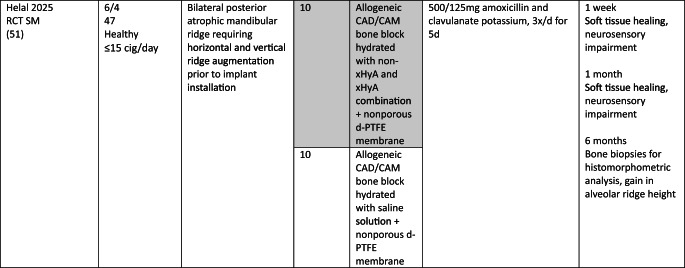
Details of the included studies listed per surgical procedure

*ASA* American society of anesthesiologists; *β-TCP* beta-tricalcium phosphate; *C-DBBM* collagenated demineralized bovine bone mineral; *CAD* computer aided design; *CAM* computer aided manufacturing; *CHBG* collagenated heterologous bone graft; *cig* cigarettes; *CT* controlled trial; *d-PTFE* dense polytetrafluorethylene; *DBBM* demineralized bovine bone mineral; *DFDBA* human demineralized freeze-dried bone allograft; *FGG* free gingival graft; *HyA* hyaluronic acid; *i.v.* intravenous; *I-PRF* injectable platelet rich fibrin; *NR* not reported; *OHIP* oral health impact profile; *PG* parallel group; *RCT* randomized controlled trial; *SM* split mouth; *SMI* structural model index; *SWHS* socket wound healing score; *URPF* ultrasonic resorbable pin fixation; *xHyA* crosslinked hyaluronic acid

Elian et al. [52]

### Study intervention

All details of the performed interventions are presented in Table 1.

In 4 MSFA studies, HyA was either applied as gel alone [30], or as gel mixed with a BSM [31, 46, 47]. Further, in 3 MSFA studies a pre-mixed putty material consisting of HyA and a BSM was used, alone [29] or additionally combined with autologous bone [44, 45].

In one ARP study, HyA was applied as gel alone [32] and in one study combined with autologous bone [50]. In another 2 studies, HyA was mixed with a BSM and covered with a free gingival graft (FGG) [48, 49]. In the fifth ARP study, BSM was applied into the socket, covered with a collagen matrix, and a HyA gel was applied 3-times per day locally by the patients for 7 days [33].

In one GBR study, HyA gel was mixed with a BSM and covered with a resorbable collagen membrane for lateral ridge augmentation [34], while in the other study computer aided design/computer aided manufacturing (CAD/CAM) fabricated allogeneic bone blocks were soaked either in saline solution or in HyA and covered by a d-PTFE membrane for lateral and vertical ridge augmentation [51].

### HyA information

All HyA products used in the included studies were commercially available; information about the HyA formulation was collected, when available, from all possible sources (i.e., publications and internet). The concentration of HyA varied between 0.2 to 1.6%, whereas this information was not available for 3 studies [29, 44, 45]. In one study each, either only crosslinked HyA (xHyA) [49] or only non-crosslinked HyA (non-xHyA) [33] was used, while in 4 studies a combination of xHyA and non-xHyA was applied [31, 34, 48, 51]. In 3 studies the used product was already available as mixture of HyA with BSM (i.e., a putty); in 2 studies HyA was mixed with beta-tricalcium phosphate (β-TCP) [44, 45], and in one study with demineralized freeze-dried bone allograft (DFDBA) [29]. One study impregnated a CAD/CAM fabricated allogeneic bone block with a combination of non-xHyA and xHyA [51] and one study did not report on the chemical form of HyA [32]. HyA was either applied/used as gel (n = 10) [30–34, 46–48, 50, 51], putty (n = 3) [29, 44, 45], or solution (n = 1) [49]. The details about the HyA products are presented in Table 2.

**Table 2:** HyA products used in the included studies

Product (Trade name)	Producer (Manufacturer, country)	HyA concentration	Chemical form	Application form	Study (year)
CEROS® TCP-Putty	MathysMedical, Switzerland	NR	β-TCP (94%) granules embedded in a sodium HyA (6%) hydrogel matrix^1^	Putty	Stiller (2014), Knabe (2017)
DBX®	Musculoskeletal Transplant Foundation, USA	NR	DFDBA (32%) in a HyA (68%) carrier	Putty	Schwartz (2007)
Gengigel forte	Ricerfarma srl, Italy	0.8%	non-crosslinked	Gel	Eeckhout (2022)
HyadentBG	Regedent AG, Switzerland	1.6% 0.2%	1.6% crosslinked 0.2% non-crosslinked	Gel	Velasco-Ortega (2021), Husseini (2023), Kauffmann (2023), Helal (2025)
Hyaloss™ Matrix	ANIKA Therapeutics, USA	20-60mg/ml	20 – 60mg/ml esterified HyA fibers	Matrix/Gel	Baldini (2010), Göcmen (2016), Dogan (2017), Gurbuz (2022)
Nikkol	BS Pharma, Brazil	1%	NR	Gel	Alcantara (2018)
Perfectha	Sinclair Pharma GmbH, Germany	20 mg/ml	crosslinked	Solution	Abaza (2024)

*β*-TCP beta-tricalcium phosphate; *DFDBA* demineralized freeze-dried bone allograft; *HyA* hyaluronic acid; *NR* not reported

^1^This information was extracted from: Bohner [53]

### Clinical setting and funding details

Eleven studies were performed in a single university department [29–31, 34, 44–49, 51], and 2 studies were performed as multicenter studies [32, 33]; the information was unclear in one study [50].

Three studies reported receiving funding from their respective universities [33, 46, 47], while 5 studies reported receiving grants from external funding organizations [29, 32, 44, 45, 51]. Further, one study reported not having received any financial support [34] and 5 studies have not given any information about any funding [30, 31, 48–50]. In 3 of the above listed studies, the products used were additionally provided by the manufacturers [29, 31, 33].

### Reported outcome variables and follow-up

The following summary focuses on the pre-defined primary and secondary outcome parameters. For further details on additional parameters see Table 3 (MSFA), 4 (ARP), and 5 (GBR).

**Table 3:**
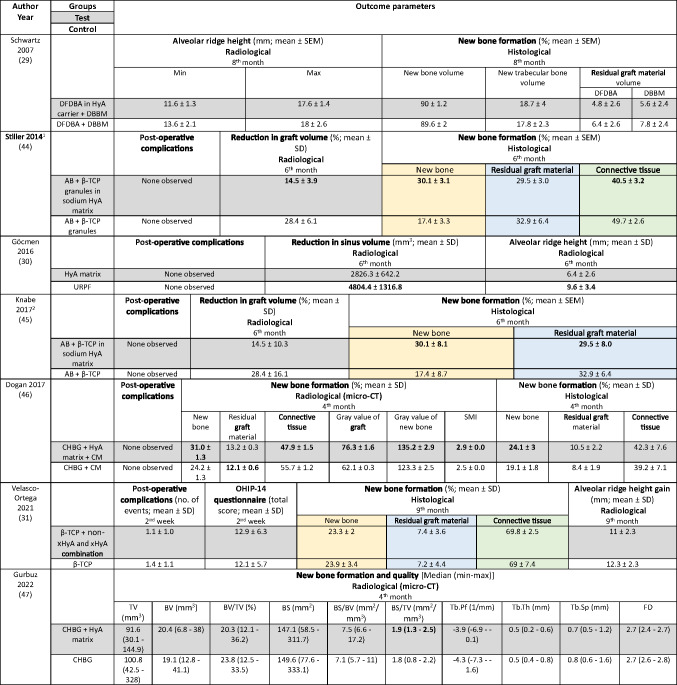
Clinical, histological, and/or radiographic results of patients treated with a maxillary sinus floor augmentation procedure; only relevant groups allowing to assess the effect of HyA are listed. Significant differences between the control and test group are highlighted in bold for the superior group. RCT applying somehow comparable surgical techniques and materials and had a comparable follow-up time and outcome assessment are highlighted in the same colors; please note that due to relevant differences in augmentation material and HyA product in the 3 highlighted studies, meta-analysis was deemed not appropriate

*AB* autologous bone; *β-TCP* beta-tricalcium phosphate: *BS* bone surface; *BS/BV* specific bone surface; *BS/TV* bone surface density; *BV* bone volume; *BV/TV* bone volume fraction; *CHBG* collagenated heterologous bone graft; *CM* collagen membrane; *Col* 1 collagen Type 1; *FD* fractal dimension; *HyA* hyaluronic acid; *Max* Maximum; *Min* minimum; *NR* not reported; *OHIP* Oral Health Impact Profile; *SD* standard deviation; *SEM* standard error of the mean; *SMI* structural model index; *Tb.Pf* trabecular pattern factor; *Tb.Sp* trabecular separation; *Tb.Th* trabecular thickness; *TV* total volume; *URPF* ultrasonic resorbable pin fixationz; *xHyA* crosslinked hyaluronic acid

^1^Please note that the immunohistochemical analysis is not listed here; however, no significant differences have been detected between the control and HyA group in any of the assessed parameters

^2^Please note that the immunohistochemical analysis is not listed here; for details including a few significant differences please refer to the original.

The MSFA studies (Table 3) had a follow-up of 4 to 9 months. Two studies reported the radiologically assessed ARH [29, 30], and one study the ARH gain [31]. Four studies analyzed NBF in bone biopsies histologically [29, 31, 44, 45], one study with micro-CT [47], and one study histologically and with micro-CT [46]. Five MSFA studies reported on post-operative complications [30, 31, 44–46], and one study analyzed PROM [31].

In the ARP studies (Table 4) the hard tissue healing was evaluated after 3 to 4 months. In 2 studies ARW was measured radiologically [32, 33], and in one study it was measured both clinically and radiologically [49]. One study [48] assessed radiologically volumetric and linear resorption of the entire alveolar ridge, which represented a combination of any changes in width and height. NBF was measured radiologically in one study [32] and histologically in 2 studies [49, 50]. Post-operative complications were evaluated in 2 studies [49, 50] and another study assessed early wound healing and pain [33].

**Table 4:**
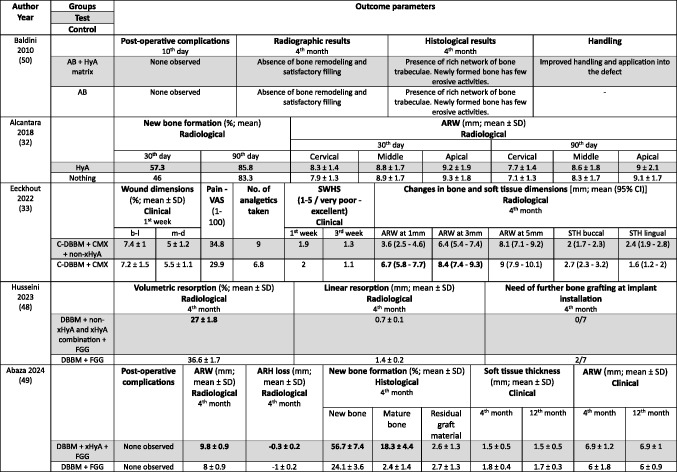
Clinical, histological, and/or radiographic results of patients treated with alveolar ridge preservation; only relevant groups allowing to assess the effect of HyA are listed. Significant differences between the control and test group are highlighted in bold for the superior group. None of the included studies applied somehow comparable techniques and materials and had a comparable follow-up and outcome assessment

*AB* autologous bone; *ARW* alveolar ridge width; *b-l* bucco-lingual; *C-DBBM* collagenated demineral confidence interval; *CMX* collagen matrix; *DBBM* demineralized bovine bone mineral; *FGG* free gin acid; m-d mesio-distal; *NR* not reported; *SD* standard deviation; *STH* soft tissue height; *SWHS* Socke visual analog scale; *xHyA* crosslinked hyaluronic acid

Both GBR studies (Table 5) evaluated hard tissue healing after 6 months and bone biopsies were taken for histological analysis. ARW was measured clinically in one study [34], while the other study assessed ARH gain radiologically [51]. The latter study [51] followed the patients for 12 months and assessed bucco-lingual marginal bone loss at the implants at the end of the follow-up.

**Table 5:**
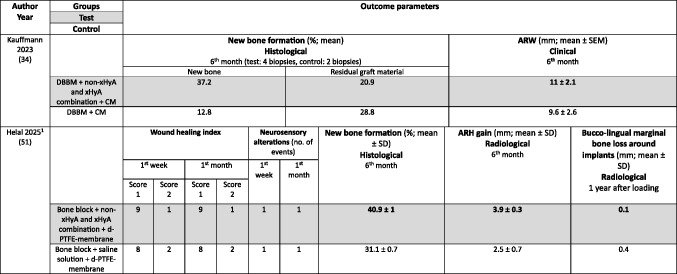
Clinical, histological, and/or radiographic results of patients treated with guided bone regeneration for lateral and/or vertical alveolar ridge augmentation; only relevant groups allowing to assess the effect of HyA are listed. Significant differences between the control and test group are highlighted in bold for the superior group. None of the included studies applied somehow comparable techniques and materials and had a comparable follow-up and outcome assessment

*ARH* Alveolar ridge height; *ARW* Alveolar ridge width; *CI* confidence interval; *CM* collagen membrane; *DBBM* demineralized bovine bone mineral; *HyA* hyaluronic acid; no. number; *d-PTFE* dense polytetrafluorethylene; *SD* standard deviation; *SEM* Standard error of the mean; *xHyA* crosslinked hyaluronic acid

^1^Please note, that the immunohistochemical analysis is not listed here; for details regarding the significant results please refer to the original publication

### Summary of the results of the individual studies

The following chapter concentrates again on the pre-defined primary and secondary outcome parameters. For further details on additional parameters see Table 3 (MSFA), 4 (ARP), and 5 (GBR).

In MSFA (Table 3), 3 out of 6 studies showed a significantly higher amount of NBF in the HyA group compared to the control group (2 based on histology and 1 based on histology and micro-CT) [44–46]. Further, one out of 5 studies measuring the amount of residual graft material observed significantly less residual graft material in the HyA group [45]. Another study reported conflicting results regarding the impact of HyA on graft resorption; significantly less residual graft material was observed in the control group based on micro-CT analysis but there was no significant difference in the histological analysis [46]. Two studies did not show any significant difference between the groups regarding all hard tissue parameters assessed histologically [29, 31]. One out of 3 studies measuring connective tissue content showed significantly less connective tissue in the HyA group [44], another study showed significantly less connective tissue in the HyA group based on micro-CT analysis but no difference based on histology [46], while the third study showed no significant difference [31]. In regard to post-operative ARH and ARH gain, no significant difference was observed between the groups in 2 studies [29, 31], while in one study a significantly higher ARH was observed in the control compared to the HyA group [30]. In 4 studies no post-operative complications were observed [30, 44–46], while the only study reporting complications showed no difference between the groups as well as no differences regarding self-reported oral-health related quality of life [31].

In the ARP studies (Table 4), NBF was analyzed in 3 studies [32, 49, 50], but one study performed no quantitative assessment; in this specific study a “rich network of bone trabeculae” was described for both groups [50]. NBF was significantly higher in the HyA compared to the control group after 30 days, but the difference between the groups lacked significance after 90 days in one study [32]. In the third study, the HyA group achieved a significantly higher NBF compared to the control group [49]. One study analyzed residual graft material, but detected no significant difference between the groups [49]. In 1 out of 3 studies a significantly higher ARW was observed for the test group, but only based on the radiological and not on the clinical measurements [49]; in 1 study the ARW was significantly better in the control group at the first and third mm [33] and the third study did not show any differences [32]. One additional study reported significantly less volumetric resorption of the alveolar ridge (i.e., a combination of the changes in width and height) for the test group [48]. Post-operative wound healing was recorded in one study, but no significant difference was observed between the groups [33]. In 2 studies post-operative complications were considered, but nothing was observed [49, 50].

In one GBR study (Table 5) the HyA group showed an almost 3-times higher NBF and about 30% less residual graft material compared to the control group, but due to the limited numbers of biopsies taken no statistical analysis was performed [34]. In the second GBR study a statistically significant increase in NBF was observed compared to the control group [51]. In one study the clinically assessed mean ARW was about 1.5 mm more in the HyA group reaching a statistical significant advantage compared to the control group [34], and in the second study the radiological analysis indicated an additional and statistically significant ARH gain of 1.4 mm in the test compared to the control group [51].

### Synthesis of the results

Due to the high heterogeneity in treatments and outcome measures, as well as the limited number of studies, a meta-analysis was not feasible for any of the primary or secondary outcomes.

### RoB assessment

Five of the 12 RCT showed low RoB [31, 33, 48, 49, 51], while 7 showed some concerns [30, 32, 34, 44–47]. Four studies showed some concerns in the randomization process [30, 44, 45, 47], one study showed some concerns regarding missing outcome data [46], and 3 studies showed some concerns in the selection of reported results [32, 34, 46]. No study deviated from the intended intervention, and all studies had a low RoB regarding measurement of the outcome data. One CT showed moderate overall RoB [29], while the other CT was deemed with serious overall RoB [50]. The detailed RoB analyses are presented in Appendix 5 and 6.

## Discussion

The present systematic review aimed to assess whether the application of HyA alone or in combination with additional products/carriers, such as BSM, improves hard tissue healing and/or reduces post-operative complications in alveolar bone augmentation procedures. Fourteen CT or RCT assessing the impact of HyA either in MSFA, ARP or GBR procedures were identified. The results in general indicated some positive effect of the use of HyA in MSFA, ARP, and GBR; however, the results were not always consistent and the evidence in general is limited/obscure. More specifically, the results showed that addition of HyA significantly improved NBF – at some timepoint during follow-up – in 50 and 66% of the MSFA and ARP studies, respectively; however, clinical improvements were not consistently observed. In one of the 2 GBR studies, including mainly descriptive data, the HyA group presented with about 3-times higher NBF values, about one third less residual graft material, and an increased ARW [34]; in the second GBR study significant improvements with approximately one third more NBF and about 1.5 mm more ARH gain in the test/HyA group were observed [51]. No differences were observed regarding post-operative complication rates in any of the studies.

In this context, 2 narrative [54, 55] and 2 systematic reviews, one with [27] and one without [28] a meta-analysis, exist already on this topic; however, these cover different aspects. The narrative reviews considered either only studies on ARP procedures [54] or a wide range of in vitro, preclinical, and clinical trials [55]. One of the systematic reviews also considered only ARP procedures [28], while the other one [27] concluded, based on the results of a meta-analysis, that the available data on the effect of HyA on hard tissue healing remain inconclusive. However, it should be noted that this specific meta-analysis included 3 studies, which performed 2 different procedures, i.e., MSFA and ARP [27]. Due to the significant biological and clinical differences between these 2 procedures, a meta-analysis was not performed herein. In this context, the present systematic review aimed to provide a comprehensive overview of all available clinical trials on alveolar bone augmentation procedures assessing the effect of HyA and providing a control group allowing to judge the effect of HyA. To this date, the available evidence is highly heterogeneous regarding the treatments and assessed outcomes, as well as there is an overall limited number of RCT, which in turns prevents any meaningful meta-analysis.

Most evidence on the effect of HyA on bone regeneration is from MSFA procedures, i.e., in 7 out of 14 included studies. Nevertheless, HyA was used quite differently among the studies ranging from only a gel without any other solid material for space maintenance to mixtures with BSM and/or autologous bone. Altogether, HyA showed positive effects on graft consolidation and maturation with the HyA group being superior for at least one of the relevant parameters, i.e., amount of NBF, residual graft material, and/or content of connective tissue in about half of the studies [44–46]. While these parameters supposedly improve osseointegration of dental implants and/or shorten the required healing time until implant installation, they do not necessarily affect the stability of the grafted volume and ARH gain. For example, although reduction in graft volume was significantly less (twofold) in the HyA compared to the control group in one study [44], the other MSFA studies did not show significant difference between test and control group regarding graft volume and ARH changes. Interestingly, one of the studies even showed the opposite effect with the control group being superior to the HyA group regarding graft volume and ARH [30]. However, this specific study used HyA fibers without any additional BSM and thereby indicates indirectly that HyA should probably be used in combination with a scaffold, such as BSM or autologous bone, and not as the only “grafting” material. One of the included MSFA studies [29], tested an HyA carrier in 3 different combinations, i.e., DFDBA in HyA carrier, DFDBA in HyA carrier and DBBM, and DFDBA in HyA carrier and β-TCP, but only one control group allowing to assess the effect of HyA (i.e., DFDBA + DBBM) was available. It was observed that the combination of ß-TCP with DFDBA in a HyA carrier led to significantly inferior NBF and new trabecular bone compared to the other groups, while the histomorphometric results in the other 3 groups showed no significant differences. However, any histomorphometric differences between the groups did not result in differences regarding ARH [29]. Altogether, although some positive results have been described for the adjunct use of HyA in MSFA procedures, no strong clinical recommendation can be provided and no comparison between HyA and other biologics was identified.

Five studies assessed the effect of HyA on hard tissue healing in ARP procedures. However, as for the MSFA studies, the methods to apply HyA varied, i.e., from applying only a HyA gel or mixed with autologous bone or BSM into the socket to HyA being applied topically onto the socket by the patient. The achieved bone quality (i.e., NBF) was assessed quantitatively in only 2 studies [32, 49], both indicating advantages for the application of HyA, even if in one of the studies [32] the difference was statistically significant only in the early wound healing period. Considering the clinical efficacy of the procedure, i.e., preservation of the alveolar ridge dimension, 4 studies [32, 33, 48, 49] contributed with either linear or volumetric data. Two studies [48, 49], both mixing HyA with a BSM and covering it with a FGG, reported significant advantages for the application of HyA, while one study [32] reported no difference and one study [33] even the opposite effects in the coronal aspects of the ridge. Notably, the study reporting opposite effects relied on the sole topical application of the HyA product by the patients, and HyA was not applied into the extraction socket. Unfortunately, only a single study [48] reported on one of the most relevant parameters in clinical practice after performing ARP, i.e., the need of further bone grafting at implant installation. While in the control group, about 30% of the sites required re-grafting, none of the sites treated in the HyA group required re-grafting. A recent retrospective study confirmed the potential advantages of mixing BSM with HyA in ARP procedures with significantly less ARH loss, a smaller graft shrinkage rate, and a higher bone density 4 months after performing ARP compared to a group using BSM only [56]; notably, also in this study the socket entrance was covered, not with a FGG but with a platelet-rich fibrin (PRF) plug. This might imply the need for covering the socket entrance to prolong the positive effect of HyA. One of the included studies provided also a direct comparison between HyA and another biologic, i.e., injectable PRF (i-PRF), showing superior results for HyA [49]. More specifically, after 4 months of healing, the combination of DBBM with HyA exhibited the highest ARW and the lowest ARH loss, followed by the mixture of DBBM with i-PRF and the DBBM-only group. While the i-PRF and HyA groups showed no statistically significant difference regarding ARW, they were both superior to the DBBM-only group. In addition, the HyA group was significantly superior to both other groups regarding changes/loss in ARH. Additionally, the combination of DBBM with HyA resulted in a significantly higher NBF compared to both other groups (i.e., DBBM alone and DBBM with i-PRF) as well as in significantly less residual graft material compared to the group with DBBM and i-PRF [49]. The overall positive clinical data supporting the application of HyA in ARP are supported by data derived from preclinical in vivo studies [57, 58]. In particular, one study [57] assessing the healing after ARP of compromised extraction sockets in beagle dogs reported superior histological results (i.e., regarding bone formation and maturation) for the groups with adjunctive HyA, either combined with collagenated DBBM or an absorbable collagen sponge (ACS), compared to the respective control groups without HyA. Notably, the groups with collagenated DBBM outperformed the groups with ACS regarding stability of the alveolar ridge dimension. In another study [58] the effect of HyA on the healing after ARP of infected extraction sockets in beagle dogs was assessed and compared to the effect of rhBMP-2. Both groups with an adjunct (i.e., HyA or rhBMP-2) showed superior bone healing compared to the control groups and the effect of HyA and rhBMP-2 was very well comparable.

Two RCT on assessing the effect of HyA in GBR procedures were identified herein [34, 51]. However, these 2 RCT applied quite different methods for different indications. More specifically, while one used particulated DBBM (with or without HyA) and covered the site with a resorbable collagen membrane for lateral ridge augmentation [34], the other study used allogeneic bone blocks alone or hydrated with HyA, and covered with a non-resorbable membrane for lateral and vertical ridge augmentation [51]. Independent of the different techniques and materials used, both studies reported a significant positive effect on the final alveolar ridge dimension due to the adjunctive use of HyA, i.e., either the ARW [34] or the ARH [51] was significantly wider/higher by about 1.5 mm compared to the control group. In fact, these studies reported that the quality of the augmented bone was also improved, i.e., both studies reported superior data regarding NBF compared to the control group and both studies achieved in the test groups after 6 months approximately 40% NBF. In addition, one of the studies [51] reported one year after functional loading in the HyA group also a more stable marginal peri-implant bone level, and immunohistochemistry also revealed a significantly higher vascular endothelial growth factor (VEGF) expression; this may explain the enhanced and accelerated healing observed in the HyA-activated blocks. As a reference, one RCT comparing collagen membranes with titanium meshes in GBR with DBBM (no HyA used) achieved after 6 months around 28% NBF in both groups [59]. The promising data on HyA in GBR procedures are supported by another prospective case series [60], where DBBM was mixed with HyA including a mixture of polynucleotides for lateral ridge augmentation in 6 patients. After 5 months of healing, the core biopsies from 6 implant sites showed about 40% NBF, a value similar to what reported in the CT study included herein [34, 60]; nevertheless, a much lower ARW gain was reported (i.e., approximately 4.9 versus 7.9mm) [34, 60]. In this context, preclinical studies have also demonstrated that the combination of DBBM with HyA performs better than the use of DBBM only [25, 61]. In one trial in rabbits, the mixture of DBBM with HyA resulted in a significantly increased NBF and significantly lower residual graft material in critical-sized defects compared to DBBM only [61]. Similar results were observed in another study on the healing of critical-sized defects in rats with the mixture of DBBM with HyA improving NBF compared to DBBM only [25]. In addition, this study compared HyA in a low-viscosity crosslinking agent with a group with HyA in a high-viscosity cross-linking agent; the latter achieved even better results, emphasizing the importance of reporting in detail on the characteristics of the HyA product used.

Soft tissue healing was evaluated in one ARP [33] and one GBR study [51]. In the ARP study no clear differences were detected [33], while in the GBR study one patient in the HyA group presented with minor erythema and gingival edema, but 2 patients in the control group had a flap dehiscence. However, in both cases no suppuration occurred and the complication was successfully treated with local debridement and daily irrigation [51]. Moreover, PROM were recorded in one MSFA [31] and one ARP study [33] with no significant differences between the groups. In 7 out of 14 included studies, post-operative complications were assessed [30, 31, 44–46, 50, 51]. In 5 out of these 7 studies none were observed [30, 44–46, 50], while one GBR study observed one complication per group (i.e., neurosensory alterations) [51] and one MSFA study [31] reported the mean number of events being 1.1 and 1.4 in the HyA and control group, respectively, but not being statistically significant from each other. With all studies having none or a similar number of complications in both groups, the use of different HyA products seems safe and not associated with adverse events.

**Fig. 2:**
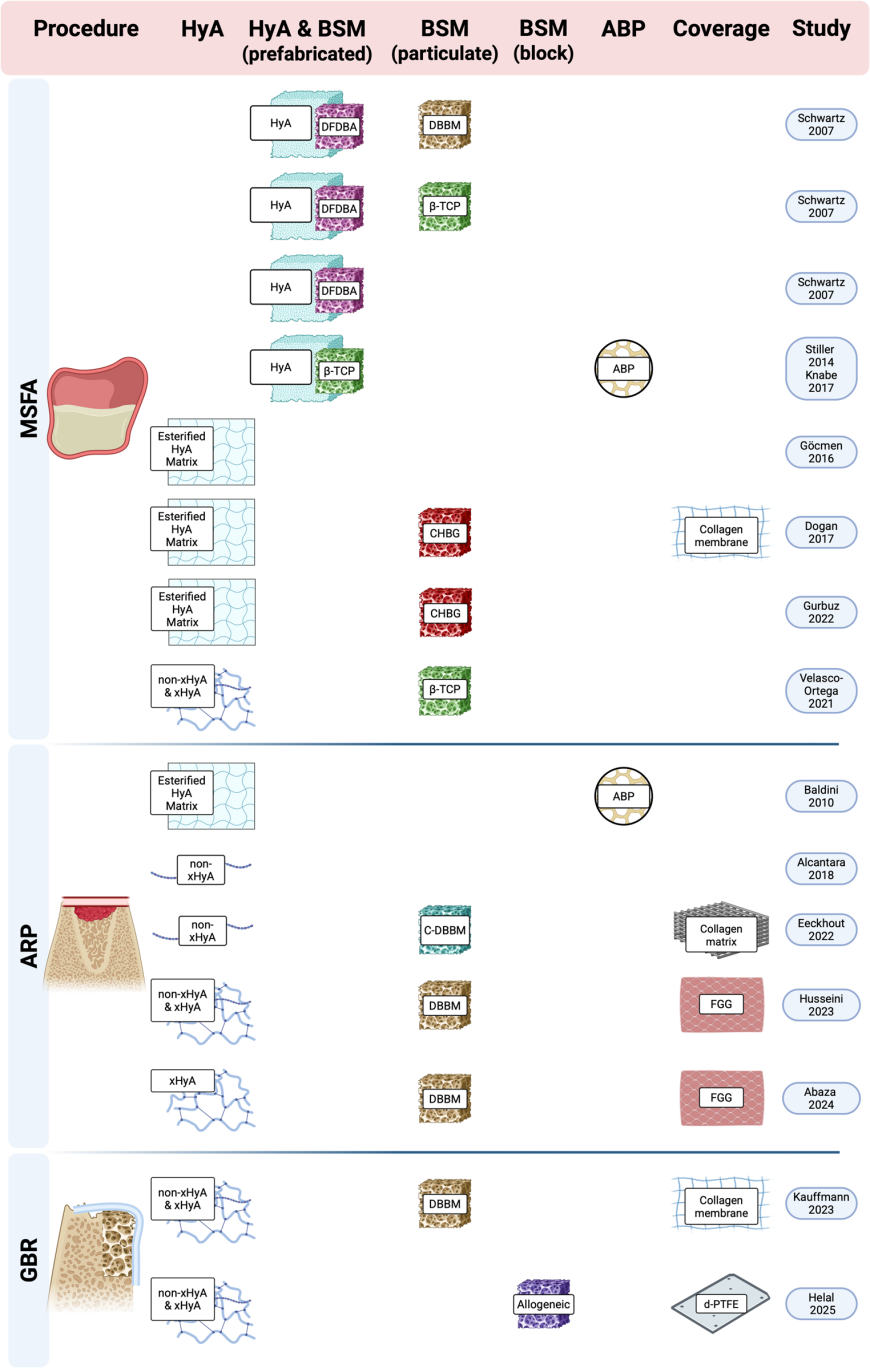
Illustration of the different approaches and HyA products used throughout the studies and procedures, which displays the observed variability among the available studies. Abbreviations: ABP—autologous bone particles; ARP—alveolar ridge preservation; BSM—bone substitute material; C-DBBM—collagenated demineralized bovine bone mineral; CHBG—collagenated heterologous bone graft; d-PTFE—dense polytetrafluorethylene membrane; DBBM—demineralized bovine bone mineral; DFDBA—demineralized freeze-dried bone allograft; FGG—free gingival graft; GBR—guided bone regeneration; HyA—hyaluronic acid; MSFA—maxillary sinus floor augmentation; non-xHyA—non-crosslinked hyaluronic acid; xHyA—crosslinked hyaluronic acid; ß-TCP—beta-tricalcium phosphate. Created in BioRender. Domic, D. (2025) https://BioRender.com/s0ub3uw

In perspective, the current evidence is limited by the large heterogeneity among the available RCT and CT, which does not allow any quantitative synthesis of the data. Specifically, variability in grafting materials, the different chemical forms and concentrations of HyA, various outcome parameters and follow-up durations, as well as the overall low number of RCT, prevented any meta-analysis for either the primary or secondary outcomes across the 3 surgical procedures (Fig. 2). In particular, the chemical form and concentration of HyA appear to be highly relevant; for example, both ARP studies reporting a significant advantage for the HyA group applied HyA in a crosslinked form (i.e., xHyA) [48, 49]. In this context, it has been previously reported, that HyA probably exerts different biological effects depending on its degree of cross-linking and/or molecular weight. More specifically, high molecular weight HyA provides anti-inflammatory and immunosuppressive properties, whereas low molecular weight HyA is more likely pro-inflammatory [62–64]. In addition, cross-linking of HyA molecules enhances the time of presence at the surgical site, which could theoretically result in improved bone healing. For example, a preclinical trial compared in a femoral condyle bone defect model the effect of non-xHyA and xHyA, both mixed with ß-TCP. The rate of new bone formation was significantly higher for the combination xHyA and ß-TCP compared to ß-TCP alone and the combination non-xHyA and ß-TCP [65]. However, any direct comparison of non-xHyA and xHyA within a clinical trial is not present in the literature so far. Finally, data on the long-term outcome, i.e., > 12 months, of the procedures performed with a HyA product are not available.

## Conclusion

The adjunctive use of HyA showed promising results on alveolar bone augmentation, in some individual studies. This positive effect was independent of the chosen procedures, but overall, the evidence is inconclusive. Any recommendation whether and how to use HyA in alveolar bone augmentative procedures requires more data derived from RCT. In addition, there was often a lack of information on the concentration and chemical form of the tested HyA product.

Based on the current evidence, the following summary can be given:

- The adjunctive use of HyA improved NBF, at some timepoint during the follow-up, in 50 and 66% of the MSFA and ARP studies, respectively.
- MSFA and ARP studies did not consistently show a benefit from using HyA regarding relevant clinical parameters, such as ARH or ARW.
- The effect of HyA in ARP may be improved by covering the socket entrance, e.g., with a FGG.
- The results of the few GBR studies appear promising and HyA is described to simplify the intra-operative handling of the grafting material; however, the evidence is very limited.
- The application of HyA does not affect the post-operative complication rate.
- Data on the long-term outcome of the procedures performed with a HyA product are not available.

## Figures and Tables

**Fig. 3:**
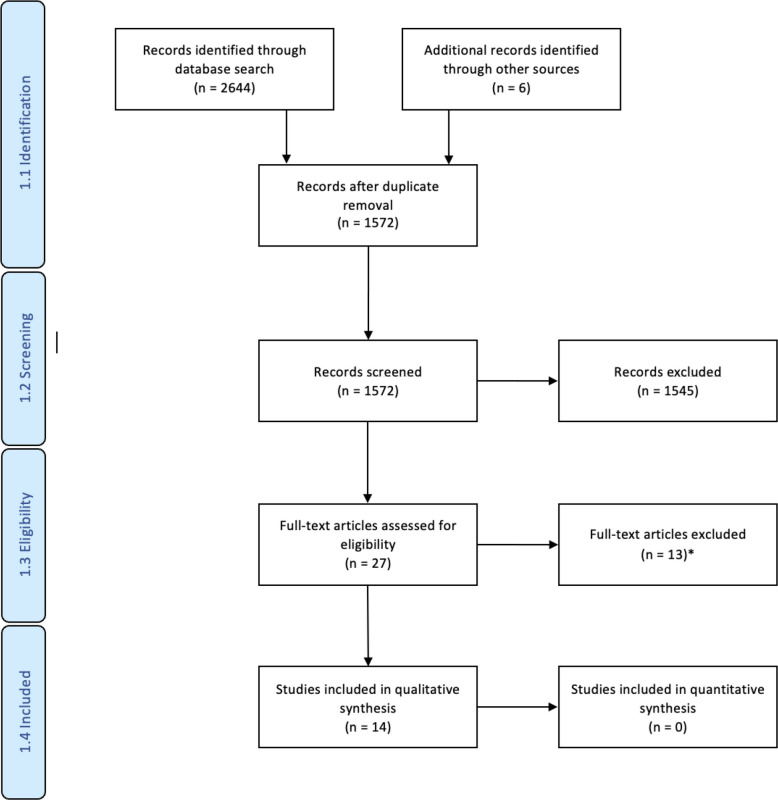
Flowchart of the literature search. * Reasons for exclusion of 13 full-text articles and the reference list are provided in Appendix 4

**Table 8:**
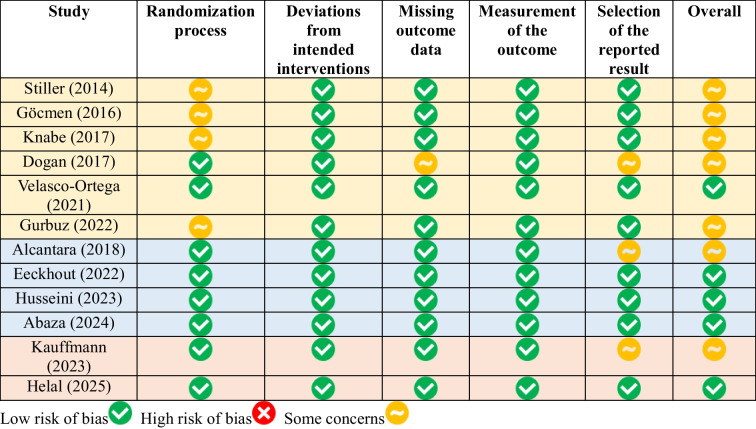
Risk of bias assessment of the randomized controlled trials according to indication: MSFA—yellow; ARP—blue; and GBR – red

**Table 9:**
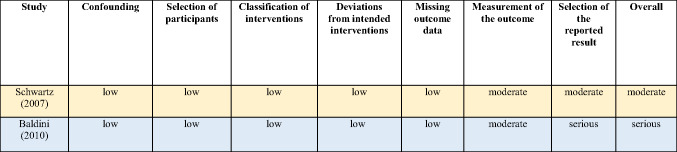
Risk of bias assessment of non-randomized controlled trials according to indication: MSFA – yellow; ARP – blue

## Data Availability

No datasets were generated or analysed during the current study.

## Appendix 1

**Table 6:** PRISMA 2020 Checklist

Section and Topic	Item #	Checklist item	Location where item is reported
**TITLE**			
Title	1	Identify the report as a systematic review.	Page 1
**ABSTRACT**			
Abstract	2	See the PRISMA 2020 for Abstracts checklist.	Page 2
**INTRODUCTION**			
Rationale	3	Describe the rationale for the review in the context of existing knowledge.	Chapter 2, page 3-4
Objectives	4	Provide an explicit statement of the objective(s) or question(s) the review addresses.	Chapter 2, page 4
**METHODS**			
Eligibility criteria	5	Specify the inclusion and exclusion criteria for the review and how studies were grouped for the syntheses.	Subheading 3.2, page 4-5
Information sources	6	Specify all databases, registers, websites, organisations, reference lists and other sources searched or consulted to identify studies. Specify the date when each source was last searched or consulted.	Subheading 3.2, page 4
Search strategy	7	Present the full search strategies for all databases, registers and websites, including any filters and limits used.	Appendix 2
Selection process	8	Specify the methods used to decide whether a study met the inclusion criteria of the review, including how many reviewers screened each record and each report retrieved, whether they worked independently, and if applicable, details of automation tools used in the process.	Subheading 3.2, page 4-5
Data collection process	9	Specify the methods used to collect data from reports, including how many reviewers collected data from each report, whether they worked independently, any processes for obtaining or confirming data from study investigators, and if applicable, details of automation tools used in the process.	Subheading 3.3, page 5
Data items	10a	List and define all outcomes for which data were sought. Specify whether all results that were compatible with each outcome domain in each study were sought (e.g. for all measures, time points, analyses), and if not, the methods used to decide which results to collect.	Tables 1, 3, 4 and 5
	10b	List and define all other variables for which data were sought (e.g. participant and intervention characteristics, funding sources). Describe any assumptions made about any missing or unclear information.	Tables 1, 3, 4 and 5, pages 6 and 12
Study risk of bias assessment	11	Specify the methods used to assess risk of bias in the included studies, including details of the tool(s) used, how many reviewers assessed each study and whether they worked independently, and if applicable, details of automation tools used in the process.	Subheading 3.4, page 5
Effect measures	12	Specify for each outcome the effect measure(s) (e.g. risk ratio, mean difference) used in the synthesis or presentation of results.	Subheading 3.5, page 5
Synthesis methods	13a	Describe the processes used to decide which studies were eligible for each synthesis (e.g. tabulating the study intervention characteristics and comparing against the planned groups for each synthesis (item #5)).	Subheading 3.5, page 5
	13b	Describe any methods required to prepare the data for presentation or synthesis, such as handling of missing summary statistics, or data conversions.	Subheading 3.5, page 5
	13c	Describe any methods used to tabulate or visually display results of individual studies and syntheses.	Tables 1, 3, 4 and 5
	13d	Describe any methods used to synthesize results and provide a rationale for the choice(s). If meta-analysis was performed, describe the model(s), method(s) to identify the presence and extent of statistical heterogeneity, and software package(s) used.	Subheading 3.5, page 5
	13e	Describe any methods used to explore possible causes of heterogeneity among study results (e.g. subgroup analysis, meta-regression).	-
	13f	Describe any sensitivity analyses conducted to assess robustness of the synthesized results.	-
Reporting bias assessment	14	Describe any methods used to assess risk of bias due to missing results in a synthesis (arising from reporting biases).	Subheading 3.4, page 5
Certainty assessment	15	Describe any methods used to assess certainty (or confidence) in the body of evidence for an outcome.	-
**RESULTS**			
Study selection	16a	Describe the results of the search and selection process, from the number of records identified in the search to the number of studies included in the review, ideally using a flow diagram.	Appendix 3
	16b	Cite studies that might appear to meet the inclusion criteria, but which were excluded, and explain why they were excluded.	Appendix 4
Study characteristics	17	Cite each included study and present its characteristics.	Tables 1, 3, 4 and 5
Risk of bias in studies	18	Present assessments of risk of bias for each included study.	Subheading 4.9, page 15
Results of individual studies	19	For all outcomes, present, for each study: (a) summary statistics for each group (where appropriate) and (b) an effect estimate and its precision (e.g. confidence/credible interval), ideally using structured tables or plots.	Subheading 4.7, page 14-15
Results of syntheses	20a	For each synthesis, briefly summarise the characteristics and risk of bias among contributing studies.	Subheading 4.7 and 4.9, page 14-15
	20b	Present results of all statistical syntheses conducted. If meta-analysis was done, present for each the summary estimate and its precision (e.g. confidence/credible interval) and measures of statistical heterogeneity. If comparing groups, describe the direction of the effect.	Subheading 4.7, page 14-15
	20c	Present results of all investigations of possible causes of heterogeneity among study results.	-
	20d	Present results of all sensitivity analyses conducted to assess the robustness of the synthesized results.	-
Reporting biases	21	Present assessments of risk of bias due to missing results (arising from reporting biases) for each synthesis assessed.	Appendix 5 and 6
Certainty of evidence	22	Present assessments of certainty (or confidence) in the body of evidence for each outcome assessed.	-
**DISCUSSION**			
Discussion	23a	Provide a general interpretation of the results in the context of other evidence.	Chapter 5, page 21-25
	23b	Discuss any limitations of the evidence included in the review.	Chapter 5, page 25
	23c	Discuss any limitations of the review processes used.	Chapter 5, page 25
	23d	Discuss implications of the results for practice, policy, and future research.	Chapter 6, page 25
**OTHER INFORMATION**			
Registration and protocol	24a	Provide registration information for the review, including register name and registration number, or state that the review was not registered.	Subheading 3.1, page 4
	24b	Indicate where the review protocol can be accessed, or state that a protocol was not prepared.	Subheading 3.1, page 4
	24c	Describe and explain any amendments to information provided at registration or in the protocol.	-
Support	25	Describe sources of financial or non-financial support for the review, and the role of the funders or sponsors in the review.	Front page
Competing interests	26	Declare any competing interests of review authors.	Front page
Availability of data, code and other materials	27	Report which of the following are publicly available and where they can be found: template data collection forms; data extracted from included studies; data used for all analyses; analytic code; any other materials used in the review.	-

From: McKenzie et al. [66]. This work is licensed under CC BY 4.0. To view a copy of this license, visit https://creativecommons.org/licenses/by/4.0/

## Appendix 2

### Search details and keywords

**Pubmed:**

(("bone regeneration"[MeSH Terms] OR "bone regeneration"[All Fields] OR "bone substitutes"[MeSH Terms) OR "bone substitutes"[All Fields] OR "alveolar ridge augmentation"[MeSH Terms] OR "alveolar ridge augmentation"[All Fields] OR "bone transplantation"[MeSH Terms] OR "bone transplantation"[All Fields] OR "guided bone regeneration"[All Fields] OR "GBR"[All Fields] OR "onlay graft"[All Fields] OR "ridge augmentation"[All Fields] OR "Sinus Floor Augmentation"[MeSH Terms] OR "sinus lift*"[All Fields] OR "sinus augmentation*"[All Fields] OR "sinus floor augmentation"[All Fields] OR "sinus floor elevation*" [All Fields] OR "sinus graft*"[All Fields] OR "maxillary augmentation"[All Fields] OR "lateral bone augmentation"[All Fields] OR "alveolar bone augmentation"[All Fields]) AND ("hyaluron*"[All Fields] OR "hyaluronic"[All Fields] OR "hyaluronan"[All Fields] OR "hyaluronate"[All Fields] OR "hyaluronic acid"[All Fields) OR "hyaluronic acid"[MeSH Terms] OR "sodium hyaluronate")).

**Embase:**

('bone regeneration'/exp OR 'bone substitutes'/exp OR 'alveolar ridge augmentation'/exp OR 'bone transplantation'/exp OR 'guided bone regeneration' OR 'GBR' OR 'onlay graft' OR 'ridge augmentation' OR 'Sinus Floor Augmentation'/exp OR 'sinus lift*' OR 'sinus augmentation*' OR 'sinus floor augmentation' OR 'sinus floor elevation*' OR 'sinus graft*' OR 'maxillary augmentation' OR 'lateral bone augmentation' OR 'alveolar bone augmentation') AND ('hyaluron*' OR 'hyaluronic' OR 'hyaluronan' OR 'hyaluronate' OR 'hyaluronic acid' OR 'hyaluronic acid'/exp OR 'sodium hyaluronate').

**OVID (MEDLINE AND Central):**

(“bone regeneration” OR “bone substitutes” OR “alveolar ridge augmentation” OR “bone transplantation” OR “guided bone regeneration” OR “GBR” OR “onlay graft” OR “ridge augmentation” OR “Sinus Floor Augmentation” OR “sinus lift” OR “sinus augmentation” OR “sinus floor augmentation” OR “sinus floor elevation” OR “sinus graft” OR “maxillary augmentation” OR “lateral bone augmentation” OR “alveolar bone augmentation”) AND (“hyaluron” OR “hyaluronic” OR “hyaluronan” OR “hyaluronate” OR “hyaluronic acid” OR “sodium hyaluronate”).

## Appendix 4

**Table 7:** Reason for exclusion of 13 studies

Author Year	Title	Reason for exclusion
Engström [67]	The effect of hyaluronan on bone and soft tissue and immune response in wound healing	Treatment modality not meeting inclusion criteria
Butz [68]	Sinus augmentation with bovine hydroxyapatite/synthetic peptide in a sodium hyaluronate carrier (PepGen P-15 Putty): a clinical investigation of different healing times	No control group available allowing to assess the effect of HyA
Emam [69]	Microcomputed tomographic and histologic analysis of anorganic bone matrix coupled with cell-binding peptide suspended in sodium hyaluronate carrier after sinus augmentation: a clinical study	No control group available allowing to assess the effect of HyA
Emam [70]	Three-dimensional analysis of ABM/ P- 15 with sodium hyaluronate carrier in sinus augmentation	No full-text available
Emam [71]	The efficacy of a tissue-engineered xenograft in conjunction with sodium hyaluronate carrier in maxillary sinus augmentation: A clinical study	No control group available allowing to assess the effect of HyA
Jelusic [72]	Monophasic ß-TCP vs. biphasic HA/ß-TCP in two-stage sinus floor augmentation procedures – a prospective randomized clinical trial	No assessment of HyA
Lorenz [73]	Injectable Bone Substitute Material on the Basis of β-TCP and Hyaluronan Achieves Complete Bone Regeneration While Undergoing Nearly Complete Degradation	No control group available allowing to assess the effect of HyA
Cocero [74]	Efficacy of sodium hyaluronate and synthetic aminoacids in post-extractive socket in patients with liver failure: split mouth study	Follow-up time too short to evaluate bone healing (21 days)
Mostafa [21]	Effect of Hyaluronic Acid Gel on Healing of Simple Dental Extraction Sockets: A Pilot Study	Follow-up time too short to evaluate bone healing (10 days)
Blaskovic [75]	Evaluation between Biodegradable Magnesium Metal GBR Membrane and Bovine Graft with or without Hyaluronate	Study design not matching inclusion criteria (retrospective study)
Beretta [60]	Xenograft combinbeed with a mixture of Polynucleotides and Hyaluronic Acid (PNs-HA) for Horizontal Alveolar Bone Regeneration: Clinical and Histological Assessments in a Case Series	Study design not matching inclusion criteria (case series)
Kloss [56]	Enhanced alveolar ridge preservation with hyaluronic acid-enriched allografts: a comparative study of granular allografts with and without hyaluronic acid addition	Study design not matching inclusion criteria (retrospective study)
Ruggiero [76]	Hyaluronic Acid Treatment of Post-Extraction Tooth Socket Healing in Subjects with Diabetes Mellitus Type 2: A randomized Split-Mouth Controlled Study	Follow-up time too short to evaluate bone healing (21 days)

*HyA* hyaluronic acid
